# Multivariate multiscale complex network analysis of vertical upward oil-water two-phase flow in a small diameter pipe

**DOI:** 10.1038/srep20052

**Published:** 2016-02-02

**Authors:** Zhong-Ke Gao, Yu-Xuan Yang, Lu-Sheng Zhai, Wei-Dong Dang, Jia-Liang Yu, Ning-De Jin

**Affiliations:** 1 School of Electrical Engineering and Automation, Tianjin University, Tianjin 300072, China

## Abstract

High water cut and low velocity vertical upward oil-water two-phase flow is a typical complex system with the features of multiscale, unstable and non-homogenous. We first measure local flow information by using distributed conductance sensor and then develop a multivariate multiscale complex network (MMCN) to reveal the dispersed oil-in-water local flow behavior. Specifically, we infer complex networks at different scales from multi-channel measurements for three typical vertical oil-in-water flow patterns. Then we characterize the generated multiscale complex networks in terms of network clustering measure. The results suggest that the clustering coefficient entropy from the MMCN not only allows indicating the oil-in-water flow pattern transition but also enables to probe the dynamical flow behavior governing the transitions of vertical oil-water two-phase flow.

Vertical upward oil-water two-phase flow is widely encountered in petroleum industry. An oil well is vertically drilled through the ground at first, and then it goes on following an inclined angle before it finally enters into the oil reservoir. The oil and water usually coexist during the above oil-well production, and these two immiscible fluids can distribute themselves in many temporal-spatial configurations, known as flow patterns, which greatly depend on the fluid properties, volume fraction and flow rates. Note that the flow behavior underlying multiphase flow is much more complicated than that of single-phase flow, due to the influence of phase interfacial interactions and local relative movements. Characterizing the dynamic behavior governing the transitions among vertical upward oil-water flow patterns has become a challenging subject of significant importance, especially for the physical modeling and flow parameters measurement.

The investigations on complicated flow behaviors underlying two-phase flow patterns have drawn a great deal of attention from various research areas. The nonlinear time series analysis methods^1^,^2^, laser-induced fluorescence method^3^, probability density function^4^, physical model^5^, continuous hidden Markov model^6^, time-frequency representation^7^, and recurrence network^8^ have been implemented to characterize two-phase flow patterns. Despite the existing developments on the characterization of flow patterns, there still exist some significant challenges. The traditional single sensor measurement, e.g., ring-shape conductance sensor or double-helix capacitance sensor or ultrasound sensor, allows capturing the global flow behavior but ignores the local flow information which is important for further uncovering the complicated flow structure. To address this problem, distributed conductance sensors have been proposed and developed for measuring the local flow behavior at different positions. Under this research background, one key challenge is how to effectively analyze the multivariate measurements to reveal the local flow behavior accounting for the formation and transition among dispersed oil-in-water flow patterns. In this regard, developing a novel approach to fuse multivariate signals measured from distributed conductance sensor would be particularly necessary and beneficial.

In recent years, the research into complex networks has undergone a remarkable development^9^,^10^,^11^,^12^,^13^,^14^,^15^,^16^. Representing constituents as nodes and regarding the interactions between constituents as connections allows us to construct a complex network from a complex system. The successful applications of complex network in different disciplines have reflected the insight that the complex network is a powerful tool for studying complex systems. Quite recently, complex network analysis of time series elicits a great deal of interests from different research fields^17^,^18^,^19^,^20^,^21^,^22^,^23^,^24^,^25^, including brain functional networks^26^,^27^, climate networks^28^,^29^, turbulent heated jets^30^, friction networks^31^ and multiphase flows^32^,^33^,^34^, etc. Introducing complex network into multivariate information fusion allows us to analyze the distributed sensor data to uncover the complicated local flow behaviors of vertical upward dispersed oil-in-water flows.

Multivariate multiscale analyses provided us with an important perspective for characterizing complex systems^35^,^36^. We proposed a multivariate multiscale complex network (MMCN) method^25^ to analyze multivariate time series. As a further study, we in this paper develop the MMCN to reveal the complicated oil-in-water local flow behavior underlying high water cut and low velocity vertical upward oil-water two-phase flow. The vertical upward oil-water two-phase flow in a small diameter pipe presents the features of multiscale, unstable and non-homogenous and its underlying dynamical behavior is much more complicated than that of gas-liquid two-phase flow. We carry out oil-water two-phase flow experiments in a vertical upward small diameter pipe at high water cut and low velocity and measure the local flow behavior for three typical oil-water flow patterns by using our designed distributed conductance sensor. Then, we infer the MMCN from multi-channel measurements and exploit clustering coefficient entropy to characterize the local flow behavior leading to the evolutions of different vertical oil-water flow patterns. Our analysis yields deep insights into the dynamical behavior of vertical upward oil-water two-phase flow from the perspective of complex network and multiscale analysis.

## Results

### Experimental design and data acquisition

**Figure 1:**
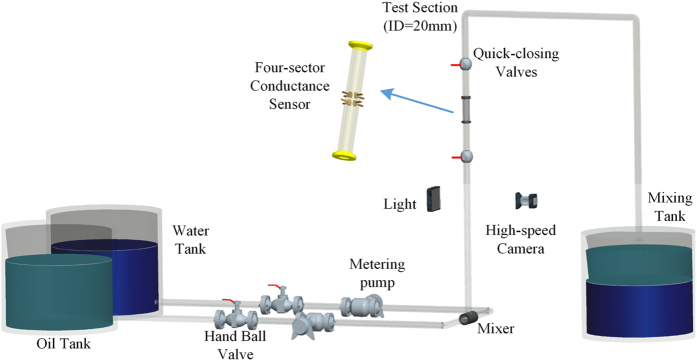
The schematic of vertical upward oil-water two-phase flow loop facility.

We carry out the oil-water two-phase flow experiment in a vertical upward small diameter plexiglass pipe (20 mm-inner-diameter) at Tianjin University. The experiential mediums are tap water and No. 3 industry white oil. Figure 1 shows the schematic diagram of the experimental flow loop. The oil-water two-phase flow loop is consisted of a water tank, an oil tank, a mixing tank, two peristaltic metering pumps, and a vertical testing pipe. During the experiment, the two phases, i.e. oil and water, are firstly pumped out from the tanks respectively, and then flow into the vertical testing pipe and eventually are drained into the mixing tank, where the two phases will separate by gravity. The peristaltic pumps used in the experiment are high-precision metering pumps, which enable to obtain the precise information of the inlet flow rate and water cut.

As shown in Fig. 1, we in this experiment install the distributed conductance sensor and high-speed camera on the vertical testing pipe. The high-speed camera enables to record and classify different flow patterns. The experimental plan is as follows: we first fix the water cut and then gradually increase the total velocity of the oil-water mixture. When the total flow rate reaches a preset value, the multivariate signals from the distributed conductance sensor are collected. The measured multivariate signals contain the information about oil-in-water local flow behaviors. In this experiment, the water cut is in the range of 80%–100%, while the mixture total flow rate is set at 0.0184 m/s, 0.0368 m/s, 0.0737 m/s, 0.1105 m/s, 0.1474 m/s, 0.1842 m/s, 0.2210 m/s and 0.2579 m/s respectively. The sampling rate is 4 kHz and the sampling duration for each measurement is 30 s. The multivariate signals from distributed conductance sensor are recorded by National Instrument Corporation’s data acquisition card PXI 4472 under LabVIEW operating environments.

### MMCN analysis of vertical oil-water two-phase flow

**Figure 2:**
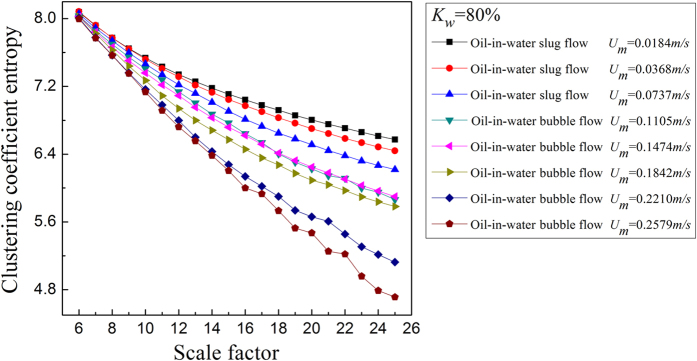
The clustering coefficient entropy of multiscale complex networks at different flow conditions when the water cut *K*_*w*_ = 80%. The distribution of the clustering coefficient entropy with the change of the scale factor.

**Figure 3:**
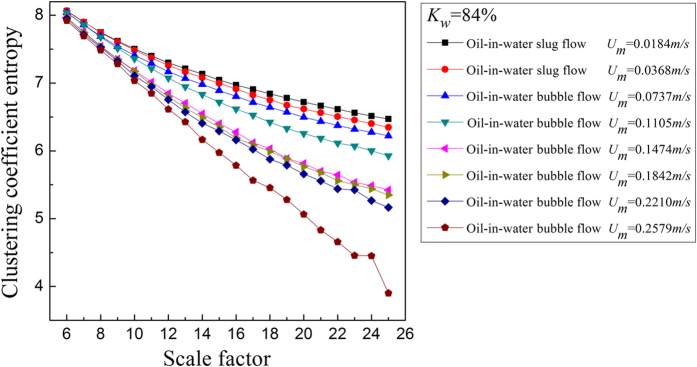
The clustering coefficient entropy of multiscale complex networks at different flow conditions when the water cut *K*_*w*_ = 84%. The distribution of the clustering coefficient entropy with the change of the scale factor.

**Figure 4:**
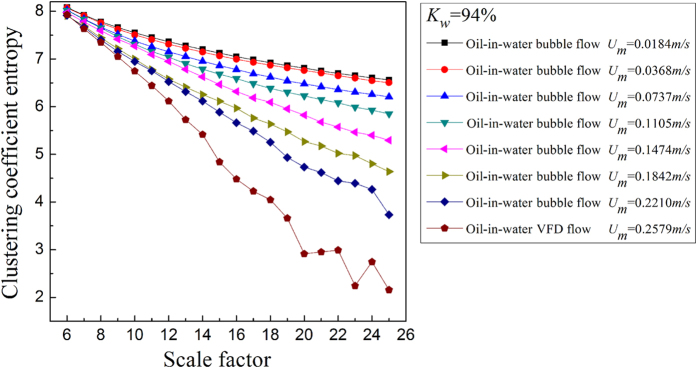
The clustering coefficient entropy of multiscale complex networks at different flow conditions when the water cut *K*_*w*_ = 94%. The distribution of the clustering coefficient entropy with the change of the scale factor.

**Figure 5:**
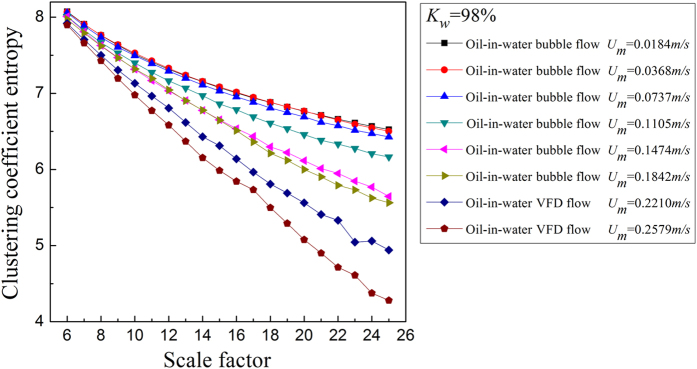
The clustering coefficient entropy of multiscale complex networks at different flow conditions when the water cut *K*_*w*_ = 98%. The distribution of the clustering coefficient entropy with the change of the scale factor.

Our method enables to map a multivariate time series into a multiscale complex network, which allows us to investigate the inherent properties of multivariate time series from the perspective of complex network analysis and multiscale analysis. Then we use network clustering measure to characterize the inherent structure of the MMCN. The clustering coefficient^37^ of a node *v* is defined as


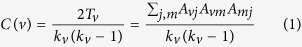


where *T*_*v*_ is the total number of closed triangles containing node *v* and *k*_*v*_ is the degree of node *v*. A large clustering coefficient indicates a specific network configuration associated with the cliquish feature of a node. According to the information entropy, we develop a novel clustering coefficient entropy, denoted as *E*_*C*_, which is calculated by:


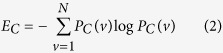



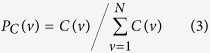


where *N* is the total number of nodes in the network. For the MMCN analysis, we calculate the *E*_*C*_ under different scales and then plot *E*_*C*_ with changing scale factors to uncover the multiscale features of the MMCN. Aiming to reveal the dynamic flow behavior in the evolution of oil-in-water flow patterns, we derive the MMCN from experimental measurements for different flow conditions. The results are shown in Figs 2, 3, 4, 5, in which *K*_*w*_ denotes the fixed water cut and *U*_*m*_ represents the total velocity of the oil-water mixture. We can see that, the distributions of *E*_*C*_ at different scales for three oil-in-water flow patterns present distinct features. Vertical oil-in-water slug flows occur at low oil-water mixture flow rate, where there exist many oil slugs whose diameters nearly equal to the pipe diameter. The local flow behavior for this flow pattern presents the features of the slow movements, intermittent oscillation and non-homogenous distribution. In particular, some small numbers of oil droplets simultaneously follow the cap-shaped oil slugs. Correspondingly, as shown in Figs 2 and 3, the clustering coefficient entropy of the oil-in-water slug flow exhibits large values. With the increase of mixture flow rate, the turbulent energy is increased, and correspondingly the oil slugs become unstable and then are dispersed into small oil droplets, i.e., an onset of an oil-in-water bubble flow. The typical feature of oil-in-water bubble flow is that the oil phase flows in a water continuum in the form of discrete droplets. As can be seen, the clustering coefficient entropy allows indicating the oil-in-water flow pattern transitions. That is, the clustering coefficient entropy decreases in the transition from the oil-in-water slug flow to the oil-in-water bubble flow, indicating that the intermittent oscillation of oil slugs gradually disappears and the movement of oil droplets becomes faster and stochastic, and the non-homogenous distribution of the oil phase becomes weak. With a further increase in the mixture flow rate, the oil droplets are broken into even smaller oil droplets in the transition from the oil-in-water bubble flow to the very fine dispersed oil-in-water bubble flow. In this flow pattern, a large numbers of smaller oil droplets uniformly disperse in the water continuous phase and randomly flow from the bottom up. Consequently, as shown in Figs 4 and 5, the clustering coefficient entropy further decreases as the flow pattern evolves into the very fine dispersed oil-in-water bubble flow, suggesting that the local flow behavior of very fine dispersed oil-in water bubble flow becomes more stochastic and the distribution of oil phase becomes more homogenous. These interesting findings demonstrate that the MMCN allows identifying three typical vertical oil-water flow patterns and further enables to reveal local flow behaviors of different flow patterns at different scales from multi-channel measurements.

## Discussions

Characterizing complicated patterns arising from vertical upward oil-water two-phase flow is a contemporary problem of significant importance. We measure the local flow information from three vertical oil-in-water flow patterns and then develop a multivariate multiscale complex network to investigate the dynamic behavior in the transitions among different oil-in-water flow patterns from multi-channel measurements. The basic idea of the MMCN is to define temporal scales in terms of the coarse-grain process and then reconstruct the phase-space from coarse-grained multivariate time series for each scale to construct a multiscale complex network. Our results indicate that the clustering coefficient entropy at different scales allows faithfully revealing the dynamical flow behavior associated with different mixture flow rate and water cut in the evolution of different flow patterns. Bridging the MMCN analysis and oil-water two-phase flow provides deep insights into the understanding of the fluid mechanism governing the formation and transition among different vertical oil-in-water flow patterns.

## Methods

### Multivariate multiscale complex network (MMCN)

For a multivariate time series containing *p* sub-time series of equal length *L* 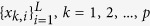, we first define temporal scales in terms of the coarse-grain process and get a coarse-grained multivariate time series^38^ in the following form:


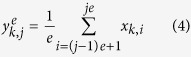


where 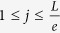, 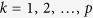 and *e* represents the scale factor. Next we derive complex networks from each 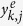 by using the multivariate embedding theory^39^. That is, we infer the complex network at different scales from multivariate time series. In particular, we reconstruct the multivariate phase-space from 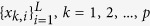 by using


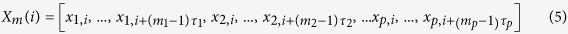


where 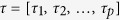 and 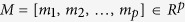 are the vector of time delay and vector of embedding dimension, respectively, and 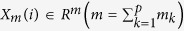. These parameters can be determined by the methods presented in Refs 25,39. Based on the above, we can infer the multivariate multiscale complex network (MMCN) as follows:

(a) We generate 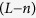 composite delay vectors 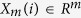, where 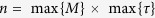 and 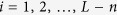; (b) We define the distance between any two phase-space vectors 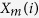 and 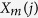, 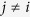 by using the maximum norm


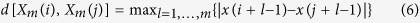


(c) We can derive a complex network by representing each phase space vector as a node and determining the connections in terms of their distances. By determining a threshold, a network adjacency matrix ***A*** can be obtained following the rule that two nodes are connected if the distance between them is smaller than the threshold: *A*_*ij*_ = 1 indicates node *i* and *j* are connected, while *A*_*ij*_ = 0 means node *i* and *j* are disconnected. The topological structure of the network can be described by the adjacency matrix ***A***. (d) Finally, we can get the MMCN by performing steps (a–c) on each coarse-grained multivariate time series. It should be pointed out that, according to Ref. 25, we normalize each sub-time series to unit variance and then use the percentage (i.e., 15%) of total variation *Tr*(*S*) to determine the threshold for constructing the MMCN, where *S* is the covariance matrix of multivariate time series.

## Additional Information

**How to cite this article**: Gao, Z.-K. *et al*. Multivariate multiscale complex network analysis of vertical upward oil-water two-phase flow in a small diameter pipe. *Sci. Rep*. **6**, 20052; doi: 10.1038/srep20052 (2016).
